# Exposure to Polycyclic Aromatic Hydrocarbons, Plasma Cytokines, and Heart Rate Variability

**DOI:** 10.1038/srep19272

**Published:** 2016-01-13

**Authors:** Binyao Yang, Qifei Deng, Wangzhen Zhang, Yingying Feng, Xiayun Dai, Wei Feng, Xiaosheng He, Suli Huang, Xiao Zhang, Xiaohai Li, Dafeng Lin, Meian He, Huan Guo, Huizhen Sun, Jing Yuan, Jiachun Lu, Frank B. Hu, Xiaomin Zhang, Tangchun Wu

**Affiliations:** 1 State Key Laboratory of Environmental Health (Incubating), School of Public Health, Tongji Medical College, Huazhong University of Science and Technology, Wuhan, Hubei, China; 2 The Institute for Chemical Carcinogenesis, The State Key Lab of Respiratory Disease, Guangzhou Medical University, Guangzhou, Guangdong, China; 3 Department of Preventive Medicine, School of Public Health, Sun Yat-sen University, Guangzhou, Guangdong, China; 4 Institute of Industrial Health, Wuhan Iron and Steel Corporation, Wuhan, Hubei, China; 5 Department of Epidemiology, Harvard School of Public Health, Boston, USA; 6 Department of Nutrition, Harvard School of Public Health, Boston, USA; 7 Channing Division of Network Medicine, Department of Medicine, Brigham and Women’s Hospital and Harvard Medical School, Boston, USA

## Abstract

Epidemiological studies have suggested associations between polycyclic aromatic hydrocarbons (PAHs) and heart rate variability (HRV). However, the roles of plasma cytokines in these associations are limited. In discovery stage of this study, we used Human Cytokine Antibody Arrays to examine differences in the concentrations of 280 plasma cytokines between 8 coke-oven workers and 16 community residents. We identified 19 cytokines with significant different expression (fold change ≥2 or ≤−2, and *q*-value <5%) between exposed workers and controls. 4 cytokines were selected to validate in 489 coke-oven workers by enzyme-linked immunosorbent assays in validation stage. We found OH-PAHs were inversely associated with brain-derived neurotrophic factor (BDNF) (*p* < 0.05), and interquartile range (IQR) increases in OH-PAHs were associated with >16% BDNF decreases. Additionally, OH-PAHs were positively associated with activated leukocyte cell adhesion molecule (ALCAM) and C-reactive protein (CRP) (*p* < 0.05), and IQR increases in OH-PAHs were associated with >20% increases in CRP. We also found significant associations between these cytokines and HRV (*p* < 0.05), and IQR increases in BDNF and CRP were associated with >8% decreases in HRV. Our results indicated PAH exposure was associated with plasma cytokines, and higher cytokines were associated with decreased HRV, but additional human and potential mechanistic studies are needed.

The increase in fossil fuel-based energy production has led to massive amounts of fine particulate matter (≤2.5 μm in aerodynamic diameter; PM_2.5_) air pollution. PM_2.5_ is a complex mixture and has been reported to be associated with increased risk of cardiovascular morbidity and/or mortality^1^,^2^. A deep insight into the constituents of particles which are responsible for these adverse cardiovascular effects is strongly needed to elucidate the mechanisms of particulate matter (PM)-related cardiovascular responses and bring forward relevant preventive countermeasures. Polycyclic aromatic hydrocarbons (PAHs) are a group of organic carbon compounds which are adsorbed on the surface of PM with high abundance, and are mostly generated from the incomplete combustion of carbon-containing materials, such as fuel and cigarette^3^. Recent epidemiologic studies consistently suggested PAH exposure is associated with elevated risks of cardiovascular diseases (CVDs)^4^,^5^.

Epidemiological studies, including those from our lab, have indicated that PAH exposure is associated with decreased heart rate variability (HRV)^6^,^7^,^8^, an established marker of cardiac autonomic dysfunction and PM-mediated cardiovascular events and mortality^9^,^10^. However, the mechanisms linking PAH exposure to reduced HRV are not completely understood. PM-induced alveolar inflammation may either directly or by the way of oxidative stress trigger systemic inflammation with accelerating blood coagulation, development of atherosclerosis, plaque instability, resulting in cardiac autonomic dysfunction and adverse cardiovascular events^1^,^11^,^12^. Toxicological evidence reported exposure of the human alveolar cell line A549 to PAHs can induce the activation of NF-κB pathway, which results in an increased interleukin-8 (IL-8) expression, and initiate or aggravate an inflammation immune response^13^,^14^. Epidemiologic evidence also revealed a link between PAH exposure and elevated levels of serum C-reactive protein (CRP)^15^,^16^. Our pilot study has examined the associations of PAH exposure with one candidate cytokine—IL-6, and of IL-6 with HRV indices, and found that IL-6 might play an important role in PAH-induced cardiac autonomic dysfunction^17^. However, the roles of other cytokines on the associations between PAH exposure and decreased HRV are still poorly understood.

Most existing epidemiological studies just have assessed associations between inflammatory markers and particulate air pollution as a whole mixture instead of specific constituents or internal exposure. In addition, some studies assessed these associations with small sample sizes^12^,^18^,^19^. Even in larger studies, these associations have been restricted to single blood measurement^20^. Thus, in the present study, we undertook a two-stage study to identify PAH-associated plasma cytokines and explore their associations with HRV through: a) comparing the cytokine expression profiles between different PAHs exposure groups using cytokine antibody array; b) validating several differentially expressed cytokines and evaluating their associations with PAH exposure; and c) assessing the associations between the PAH-associated cytokines and HRV.

## Results

### Subject characteristics

As shown in Table 1, the control group of the discovery stage was matched with the exposed group in terms of the distribution of age, gender, body mass index (BMI), waist circumference and hip circumference, waist hip ratio, seated blood pressure, smoking status, drinking status, and exercise habit (all *p* > 0.05). The distribution of these characteristics, urinary OH-PAHs, plasma cytokines, and HRV indices of 489 subjects in the validated stage were showed in Tables 1 and 2, and Table S31 (see Supplemental Material).

### Cytokine expression profiles and selected cytokines for validation

**Figure 1:**
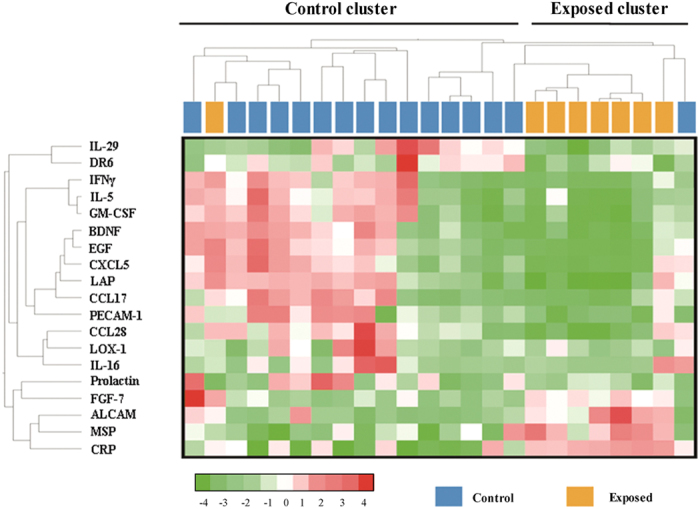
Clustering of the differentially expressed cytokines in the discovery stage. We analyzed Human Cytokine Antibody Array measurements of 280 plasma cytokines in the discovery stage with SAM to discover differences in cytokine abundance between the PAH-exposed group and the controls. We presented 19 cytokines that obtained a significant *q*-value ≤ 5% with a heat map. Samples are arranged in columns, and cytokines in rows. Red shades indicated increased expression in exposed group as compared to the controls; green shades indicated reduced expression; white shades indicated median expression. Samples are clustered into Control and Exposed categories as indicated by the first-order branches of the dendrogram (two black bars at the top).

In the discovery stage, we measured a total of 280 plasma cytokines with cytokine antibody array (Supplemental Material, Table S2), and analyzed differences of cytokine expression profiles between the exposed workers and the controls. 19 cytokines were significantly differentially expressed between the exposed group and the controls. Among these 19 cytokines, 4 were higher [all fold change (FC) ≥ 2, and *q*-value <5%] and 15 were lower (all FC ≤ −2, and *q*-value <5%) in the exposed samples (Fig. 1). We followed a serial of selection criteria (see “***Cytokine selection and validation***” in “**Materials and Methods**”) and selected 4 marked cytokines for validation from these 19 differentially expressed cytokines: brain-derived neurotrophic factor (BDNF), activated leukocyte cell adhesion molecule (ALCAM), CRP, and macrophage stimulating protein (MSP) (Table 2). Of these 4 cytokines, BDNF was lowly expressed, and ALCAM, CRP, and MSP were highly expressed in exposed samples. There are no correlations between these 4 cytokines (Supplemental Material, Table S3).

### Identification of PAH-associated cytokines

**Figure 2:**
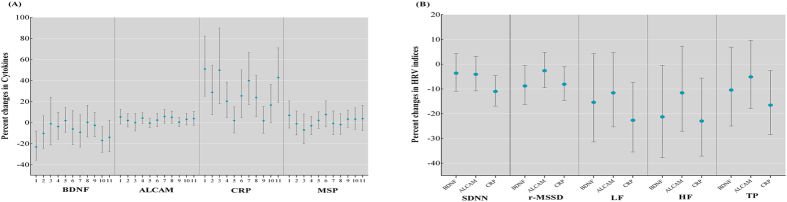
Effect estimates (percent changes with 95% CIs) for (A) plasma cytokines associated with IQR increases in the PAH metabolites: the numbers (1–11) of x-axis represent 1-hydroxypyrene, 1-hydroxynaphthalene, 2-hydroxynaphthalene, 2-hydroxyfluorene, 9-hydroxyfluorene, 1-hydroxyphenanthrene, 2-hydroxyphenanthrene, 3-hydroxyphenanthrene, 4-hydroxyphenanthrene, 9-hydroxyphenanthrene, and ΣOH-PAHs, respectively; and for (B) HRV indices associated with IQR increases of plasma cytokine levels. Models were adjusted for age, sex, BMI, smoking status, pack-years of smoking, alcohol use status, working years, and exercise.

In the present study, we observed the associations of PAH exposure and decrease in HRV indices (see Supplemental Material, Table S4). In the validation stage, we used multiple linear regression analysis to estimate the covariate-adjusted associations between creatinine-standardized OH-PAHs concentrations and 4 selected cytokines concentrations (Table 3). 1-hydroxypyrene, 9-hydroxyfluorene, and 4-hydroxyphenanthrene were negatively associated with the concentration of BDNF (all β ≤ −0.062, *p* ≤ 0.030). 1-hydroxypyrene, 1-hydroxynaphthalene, 2-hydroxyfluorene, 2-hydroxyphenanthrene, 3-hydroxyphenanthrene, and the sum of OH-PAHs (ΣOH-PAHs) were positively associated with the concentration of ALCAM (all β ≥ 0.035, *p* < 0.037). Most of the PAH metabolites, except for 9-hydroxyfluorene and 4-hydroxyphenanthrene, were positively associated with the CRP (all β > 0.108, *p* ≤ 0.02). Notably, urinary 1-hydroxypyrene was significantly associated with decreased BDNF, as well as increased ALCAM and CRP (all *p* ≤ 0.030). We also observed significant associations of OH-PAHs (without creatinine-standardized) with BDNF, ALCAM, and CRP, respectively (Supplemental Material, Table S5). Furthermore, we also analyzed the changes in cytokine concentrations associated with each interquartile range (IQR) increase in OH-PAHs (Fig. 2A). One IQR increase in 1-hydroxypyrene (3.26 μg/mmol creatinine) and 9-hydroxyphenanthrene (0.80 μg/mmol creatinine) was associated with 23.08% lower BDNF concentration [95% confidence interval (CI): −35.70% to −7.98%, *p* = 0.005] and 16.92% lower BDNF concentration (95% CI: −28.23% to −3.82%, *p* = 0.013). Whereas CRP concentration was 51.03% higher (95% CI: 25.14% to 17.76%, *p* < 0.001) per IQR increase in 1-hydroxypyrene, 28.81% higher (95% CI: 7.61% to 54.19%, *p* = 0.006) per IQR increase in 1-hydroxynaphthalene (1.43 μg/mmol creatinine), 49.81% higher (95% CI: 18.20% to 89.87%, *p* = 0.001) per IQR increase in 2-hydroxynaphthalene (1.50 μg/mmol creatinine), 20.36% higher (95% CI: 4.71% to 38.36%, *p* = 0.009) per IQR increase in 2-hydroxyfluorene (0.69 μg/mmol creatinine), 25.50% higher (95% CI: 4.88% to 50.17%, *p* = 0.013) per IQR increase in 1-hydroxyphenanthrene (1.01 μg/mmol creatinine), 39.81% higher (95% CI: 17.32% to 66.62%, *p* < 0.001) per IQR increase in 2-hydroxyphenanthrene (0.30 μg/mmol creatinine), 23.80% higher (95% CI: 5.86% to 44.77%, *p* = 0.008) per IQR increase in 3-hydroxyphenanthrene (0.36 μg/mmol creatinine), and 42.85% higher (95% CI: 19.28% to 71.06%, *p* < 0.001) per IQR increase in ΣOH-PAHs (8.15 μg/mmol creatinine) (Fig. 2A).

### PAH-associated cytokines and HRV indices

We further estimated the associations of 3 PAH-associated cytokines with HRV indices. As shown in Table 4, BDNF was associated with lower root mean of square of successive differences between adjacent normal NN intervals (rMSSD) and high-frequency (HF) power (all β < −0.029, *p* < 0.042); ALCAM was associated with lower HF (β = −0.214, *p* = 0.048); and CRP was associated with lower standard deviation of all normal to normal NN intervals (SDNN), r-MSSD, low-frequency (LF;) power, and total power (TP) (all β < −0.028, *p* < 0.039). When we further adjusted for the other 3 cytokines, the same trend of difference was observed (Table 4). We also analyzed the changes in HRV levels associated with each IQR increase in cytokine concentrations (Fig. 2B). R-MSSD and HF decreased by 8.80% (95% CI: −16.37% to −0.54%, *p* = 0.052) and 21.29% (95% CI: –37.75% to –0.48%, *p* = 0.048), respectively, in association with an IQR increase in BDNF (8.00 ng/mL). And one IQR increment in CRP (1.74 ng/mL) was associated with decreases of 11.00% (95% CI: −16.95% to −4.61%, *p* = 0.001) in SDNN, decreases of 8.09% (95% CI: −14.65% to −1.03%, *p* = 0.027) in r-MSSD, decreases of 22.67% (95% CI: −35.48% to −7.32%, *p* = 0.006) in LF, decreases of 22.98% (95% CI: −37.14% to −5.63%, *p* = 0.012) in HF, and decreases of 16.54% (95% CI: −28.47% to −2.61%, *p* = 0.021) in TP, respectively (Fig. 2B).

## Discussion

Our study has identified three PAH-associated plasma cytokines and observed their associations with decreased HRV indices, a noninvasive marker of cardiac autonomic dysfunction. We first used cytokine antibody array to compare the cytokine expression profiles between different PAH exposure groups and found 19 cytokines showing significantly differential expressions. Subsequently, in a validation study, we observed the concentration of BDNF was inversely associated with urinary OH-PAHs, while ALCAM and CRP were positively associated with urinary OH-PAHs. We also found significant negative associations between these PAH-associated cytokines and HRV indices, suggesting these cytokines might be mediators of the adverse cardiovascular effect in response to PAH exposure.

Many studies have reported associations of the exposure of environmental stimuli, including PAHs, with circulating inflammatory markers^1^,^11^,^15^, and of inflammatory markers with autonomic dysfunction and cardiovascular events^1^,^21^,^22^. Less is known about whether inflammatory markers or other plasma signaling proteins prime the process for decreased HRV in response to environmental stimuli, especially PAH exposure. Evidence revealed oxidative stress in the lungs following airborne particles exposure can induce proinflammatory cytokines, such as tumor necrosis factor-α and IL-6^23^,^24^ that can induce cardiac dysfunction and cardiovascular events^25^,^26^. PAHs are a group of pollutants of widespread concerns, and urinary PAH biomarkers were widely used for estimating human exposure to PAHs from all routes of exogenous compounds. Recent epidemiological studies have drawn special attention to the associations of urinary PAH biomarkers with inflammatory cytokines independent of smoking and other potential confounders^15^,^17^, and these biomarkers were found to be linked to decreased HRV in patients with cardiovascular conditions^27^ and healthy populations^28^.

BDNF was originally discovered in the brain as a pleiotropic peptide mediator involved in maintaining endothelial integrity and neuroplasticity and regulating physical activity^29^. We observed negative associations of PAH exposure with BDNF, which is similar to the results reported in a cohort study that indicated higher concentration of BDNF was associated with lower PAH-DNA adducts, a biomarker of PAHs exposure and DNA damage^30^. We further investigated the associations of plasma BDNF concentration with HRV indices and observed that increased levels of BDNF are associated with decreased HRV. So far, evidence from epidemiological studies regarding the association of circulating BDNF concentrations with the risk of CVD events is limited and conflicting. In a small case-control study conducted in 19 healthy controls and 31 patients with acute coronary syndrome, lower BDNF concentration was observed in patients compared with controls, indicating BDNF perhaps has a role of cardiovascular protection^31^. In contrast, higher BDNF level was found in patients with unstable angina compared with individuals with stable angina, suggesting that BDNF may play an important role in atherogenesis and plaque instability^32^. Recently, a large community-based cohort study of 3687 individuals conducted by Kaess and colleagues observed the association of higher serum BDNF with a decreased risk of CVD events and mortality, independent of standard risk factors, suggesting a causal protective role of BDNF in the pathogenesis of CVD^33^. Evidence showed conditional BDNF knockout mice with experimental infarction have greater myocardial lesions compared with wild-type mice^34^, possibly due to the function of BDNF involving in angiogenesis and vascular integrity maintaining^35^. Thus, as we observed, lower BDNF was associated with increased OH-PAHs, suggesting a protective role of BDNF. On the other hand, we subsequently found the association of higher BDNF with decreased HRV, possibly suggesting a risk role of BDNF. The conflicting results suggest a complicated biological mechanism of BDNF through which the cardiac autonomic system in response to PAH exposure.

PAH-associated ALCAM was found to be inversely associated with HRV in the present study. ALCAM also denoted CD166, belonging to the immunoglobulin gene superfamily. ALCAM is increased during inflammation and functions as a substitute for vascular cell adhesion molecule-1 during transmigration of leukocytes across central nervous system endothelium^36^. Patients with acute ischemic stroke whose plasma ALCAM concentrations were at or above the highest quartile (>46.8 ng/mL) at admission had a significantly poorer survival rate^37^. Recently, data regarding the roles of ALCAM in CVDs are not available. To the best of our knowledge, this is the first report examining the association of ALCAM level with cardiac dysfunction in response to PAH exposure, suggesting its potential pathogenic role in the development of atherosclerosis and cardiovascular events. Increased ALCAM concentrations could promote directly atherosclerosis by accelerating the recruitment of leukocytes into the microvasculature, and it maybe also contribute to inflammation and heart injury through T cell-mediated biological mechanisms^38^,^39^.

Additionally, we found the positive associations between CRP and OH-PAHs and the associations of increased CRP with decreased HRV, suggesting that individuals with systemic inflammation are more susceptible to adverse effects of PAHs on the cardiac autonomic function. Evidence has showed people exposed to high levels of ambient air PM may increase CRP levels^11^. Everett and colleagues reported associations of CRP with urinary 2-hydroxyphenanthrene and 9-hydroxyfluorene, respectively^16^. In a community-based study, individuals with >9 mg/L CRP level had a 6.1% reduction in SDNN associated with an IQR (13.4 μg/m^3^) increase in PM_2.5_ exposure^18^. Recently, a similar result showed that one IQR increment in PM_2.5_ (13.6 μg/m^3^) was associated with decrease of 24.9% in SDNN in subjects in the upper 20th percentile of CRP. Significantly stronger effects of PM_2.5_ exposure on HRV was observed among the subjects with elevated CRP levels, even at ambient exposure levels^12^.

Our study has several strengths. We have examined the associations of PAH exposure with cytokine changes and of these PAH-related cytokines with HRV in a relatively large sample size. The detection of the plasma cytokines was done in one key laboratory, and blinded duplicate samples were examined for quality assurance. In addition, we employed 10 urinary OH-PAHs as substitution for environmental PAHs exposure. Moreover, existing results from previous studies on the associations of circulating BDNF with cardiovascular events have not been consistent; our findings provide further evidence and clues to indicate the role of BDNF in the process of cardiovascular events. However, there are several limitations that should be noted when interpreting our results. First, our study is a cross-sectional study in which we measured PAH metabolites, cytokine levels, and HRV indices at the same time point, and it is not possible to determine whether differences in cytokines expression preceded or followed PAH exposures or cardiac autonomic dysfunction. Second, our sample is primarily male subjects and middle-aged which may limit generalizability of study findings. Third, three PAH-associated cytokines and other undetected cytokines in the discovery stage should be further validated in different populations with different PAH exposure levels. Fourth, we did not perform multiplicity adjustment in our study, considering False discovery rate (FDR) adjusted *p*-values sometimes is unreasonable for some data, there are two concerns with this analysis: *p*-value’s under the null hypothesis is satisfied with (1) independent and (2) following uniform (0,1) distribution. And Bonferroni correction is controversial, in part because the choice of a denominator is arbitrary (given that the set of hypotheses that should be considered is not obvious). Besides that, statistically significant correlations were not observed between OH-PAHs and between each metabolite and the sum of all OH-PAHs. Meanwhile, we also cannot eliminate the possibility that the findings from the present study are by reason of chance or bias.

## Conclusions

In summary, our study has identified three PAH-associated cytokines and found these cytokines were associated with a marker of cardiac autonomic dysfunction, suggesting that cytokines may be linked to cardiac autonomic dysfunction and may also increase vulnerability to the autonomic effects of PAHs exposure. However, further confirmatory studies and mechanistic researches are needed.

## Materials and Methods

### Study design and participants

We selected 497 participants from 1628 coke-oven workers who were employed at the coke plant of a steel mill in Wuhan (Hubei, China) for at least 1 year^6^. These individuals met the following inclusion criteria: 1) without bradyarrhythmia (heart rate <40 beats/minute) or tachyarrhythmia (heart rate >100 beats/minute); 2) without any self-reported diseases, such as cardiopulmonary diseases, chronic inflammation, kidney diseases, and cancers; 3) without any self-reported medication use in the preceding 3 months; 4) without any significant changes in their occupational experiences, living environment, and lifestyle (such as smoking and drinking) in the past 1 year; 5) can provide enough biological materials for measurement of cytokines, PAH metabolites, and creatinine. After written informed consent was obtained from each participant, trained interviewers performed a questionnaire to collect personal information such as demographic characteristics, lifestyle, medical history, medication use, occupational and environmental exposure experiences. Smoking was defined as having >1 cigarette per day for >1 year; drinking was defined as consuming alcoholic beverages at least once a week for >1 year; and physical activity was defined as regularly doing >20 min physical activity per day for >1 year. After physical examination, each participant donated ~20 mL morning urine and ~1 mL EDTA-anticoagulated overnight fasting venous blood.

The present study was divided into two stages: discovery and validation stage. As ΣOH-PAHs had the highest correlation coefficients with 10 individual urinary OH-PAHs (Supplemental Material, Table S6), we used ΣOH-PAHs as the representative PAH metabolites for sample selection for cytokine screening in the discovery stage. We selected 8 workers with higher ΣOH-PAHs among 497 workers (exposed group). In order to set up a control group with much lower PAH exposure level, we intentionally selected 16 residents without any significant occupational PAH exposure from 3053 community residents who participated in physical examinations from April 2011 to May 2011 in Wuhan^40^. These 16 residents met the above mentioned inclusion criteria, and had also provided written informed consent, personal information, and enough biological samples. They were frequency-matched with the exposed group in terms of the distribution of important characteristics, including age (±5 years), gender, BMI (±2), waist circumference and hip circumference, waist hip ratio, seated blood pressure, and lifestyle (Table 1). For these 24 subjects, we determined the concentrations of a total of 280 cytokines (Supplemental Material, Table S2) in plasma with cytokine antibody arrays, and compared the group differences of cytokine expression profiles. We then selected 4 cytokines based on a serial of criteria (see “***Cytokine selection and validation***” in “**Materials and Methods**”), and measured their concentrations in the remaining 489 workers by enzyme-linked immunosorbent assays (ELISA). For the cytokines that were significantly associated with at least one PAH metabolite, we further evaluated their associations with HRV indices. This study was reviewed and approved by the Medical Ethics Committee of Tongji Medical College, Huazhong University of Science and Technology. The methods were performed in accordance with the approved guidelines and regulations. The patients provided written informed consent before obtaining their biological materials.

### Measurement of urinary OH-PAHs and creatinine

We used gas chromatography-mass spectrometry to determine the concentrations of 12 urinary OH-PAHs: 10 were non-carcinogenic metabolites (1-hydroxypyrene, 1-hydroxynaphthalene, 2-hydroxynaphthalene, 2-hydroxyfluorene, 9-hydroxyfluorene, 1-hydroxyphenanthrene, 2-hydroxyphenanthrene, 3-hydroxyphenanthrene, 4-hydroxyphenanthrene, and 9-hydroxyphenanthrene), and 2 were carcinogenic metabolites (6-hydroxychrysene and 3-hydroxybenzo[a]pyrene) which were always below the limits of quantification and were excluded from further analyses^6^,^41^. We also measured urinary creatinine concentration using an automated clinical chemistry analyzer following Jaffe’s colorimetric method. The concentrations of OH-PAHs were calibrated by urinary creatinine and expressed as μg/mmol creatine.

### Cytokine antibody arrays

In the discovery stage, we determined the quantitative concentrations of a total of 280 cytokines (Supplemental Material, Table S2) with Human Cytokine Antibody Array Q6000 (Raybiotech Inc., Georgia, USA, www.raybiotech.com) in 8 exposed and 16 control plasma samples individually according to the manufacturer’s instructions. This quantitative array platform is a combination of seven forty-cytokine Quantibody arrays and uses the multiplexed sandwich ELISA-based technology. Briefly, multiple cytokines specific capture antibodies were first bound to glass surfaces. After incubation with the plasma samples, the target cytokines were trapped on the solid surface. Then we added biotin-labeled detection antibodies specific for different cytokines, which can recognize different isotope of the target cytokine.

### Cytokine selection and validation

We selected cytokines based on the following criteria: 1) showed at least a 2-fold higher (FC ≥ 2) or lower (FC ≤ −2) expression in the exposed group compared with the control group; 2) with highly significant differences in expression (*q* < 5.00%) between exposed and control group; 3) with signal values higher than 500 in each individuals; 4) were found to be involved in inflammation processes and/or be related to PM/PAH-mediated cardiovascular risk based on an extensive literature review. Four cytokines met these criteria: BDNF, ALCAM, CRP, and MSP. The concentrations of these four cytokines were measured by ELISA method in 489 workers in the validation stage. The concentrations of BDNF and CRP were measured using commercially available ELISA kits (R&D Systems, Minneapolis, Minn) according to the manufacturer’s instructions, and the detection limits for BDNF and CRP were 0.2 ng/mL and 0.01 ng/mL, respectively. The concentration of MSP was determined using Human MSP ELISA Kit ab100612 (Abcam, Cambridge, Mass), the detection limit for MSP was 0.8 pg/mL. The concentration of ALCAM was examined using ELISA Duoset from R&D Systems (DY656): briefly, a 96-well microplate was coated with 100 μL per well of capture antibody at a concentration of 2 μg/mL^42^.

### Measurements of HRV indices

We measured the HRV indices by 3-channel digital Holter monitors (Lifecard CF; Del Mar Reynolds Medical, Inc., Whitney, Irvine, USA) with a 1024 samples/second sampling rate for 10 minutes^6^. We analyzed two time-domain HRV indices: SDNN and rMSSD. We also analyzed three frequency-domain indices: LF (0.04–0.15 Hz), HF (0.15–0.4 Hz), and TP (approximately ≤0.4 Hz).

### Statistical analysis

We conducted data analysis using SPSS, version 18.0 (SPSS Inc., Chicago, IL, USA). The levels of 10 creatinine-adjusted non-carcinogenic OH-PAHs, four plasma cytokines, and five HRV indices were natural logarithm (ln) transformed to improve normality and stabilize variance. In the discovery stage, the differences of general characteristics between control and exposed groups were analyzed using Student’s *t*-test (for continuous variables) and chi-square test (for categorical variables). The cytokine antibody array data were analyzed using Significance Analysis of Microarray (SAM) 3.00 algorithm^43^ (http://statweb.stanford.edu/~tibs/SAM/index.html). SAM assigns a *d*-score to each cytokine on the basis of a multi-comparison analysis of expression changes and indicates significance by FC and *q*-value. In the validation stage, the associations between creatinine-adjusted urinary OH-PAHs concentrations and plasma cytokines, and between PAH-associated cytokines and HRV indices, were evaluated using multiple linear regression models with adjustment for age (continuous), gender (binary), BMI (continuous), smoking status (binary), pack-years of smoking (continuous), alcohol use status (binary), working years (continuous), and exercise (binary). Percent change in each cytokine and in each HRV index are presented with the increase of an IQR in PAH exposure levels and in cytokine concentrations, respectively, based on the following calculation formulas: [10(β × IQR) – 1] × 100%, with 95%CI {10[IQR × (β ± 1.96 × SE)] – 1} × 100%, where β and SE are the effect estimate and its standard error. We defined statistical significance as *p* value <0.05. Additionally, we have validated all these related linear regression models with linearity, no auto-correlation, no multicollinearity, normality on residuals, and homoscedasticity.

## Additional Information

**How to cite this article**: Yang, B. *et al.* Exposure to Polycyclic Aromatic Hydrocarbons, Plasma Cytokines, and Heart Rate Variability. *Sci. Rep.* **6**, 19272; doi: 10.1038/srep19272 (2016).

## Supplementary Material

Supplementary Information

## Figures and Tables

**Table 1:** General characteristics of the discovery and validation populations.

Characteristic	Discovery stage			Validation population
	Control group (n = 16)	Exposed group (n = 8)	*p*-Value^b^	n = 489
General characteristics				
Age (years)^a^	50.4 ± 6.4	48.3 ± 4.3	0.419	41.9 ± 8.3
Gender (Male/Female)	12/4	6/2	1.000	438/51
BMI (kg/m^2^, mean ± SD)^a^	25.0 ± 2.9	25.0 ± 4.9	0.986	23.9 ± 3.5
No. of working years (years)^a^	–	27.5 ± 5.3	–	21.1 ± 9.6
Waist circumference (cm)^a^	85.9 ±8.7	88.9 ± 11.5	0.480	83.4 ± 9.1
Hip circumference (cm)^a^	95.6 ± 4.7	97.8 ± 4.5	0.290	95.2 ± 7.8
Waist hip ratio^a^	0.90 ± 0.07	0.91 ± 0.08	0.784	0.87 ± 0.07
Seated blood pressure (mm Hg)^a^				
Systolic blood-pressure	134.4 ± 19.5	128.8 ± 18.1	0.499	118.9 ± 15.5
Diastolic blood-pressure	82.8 ± 11.4	85.0 ± 11.0	0.649	81.2 ± 10.8
Smoking status (Yes/No)	6/10	2/6	0.667	291/198
Pack-years of smoking^a^	21.1 ± 11.8	23.8 ± 11.6	0.785	19.0 ± 13.5
Drinking status (Yes/No)	5/11	0/8	0.130	184/305
Exercise (Yes/No)	7/9	4/4	1.000	208/276 c

^a^Values are mean ± SD.

^b^*p*-Value determined by Student’s *t*-test for continuous variables and by Fisher’s Exact Test for categorical variables.

^c^Information missing on exercise for 5 people.

**Table 2:** Concentrations and related functions of the 4 selected plasma cytokines.

Cytokines	Discovery stage		Validation stage^c^	Related functions	References
	FC^a^	*q*-value^b^ (%)			
BDNF	−3.62	<5.00	4.35 (1.75, 9.74)	Plays a protective role by inducing angiogenesis; induction of methylation changes in response to PAHs	30,34
ALCAM	2.74	<5.00	106.22 (80.64, 134.09)	Regulation of endothelial cell function; induce inflammation by recruitment of leukocytes	39,44
CRP	2.01	<5.00	1.14 (0.45, 2.20)	Induction of proinflammatory effects via NF-κB activation;associated with cardiovascular risk factors in response to particulate matter	12,45
MSP	2.31	5.00	93.52 (56.49, 153.49)	Plays a dual role in regulating inflammation	46

^a^Negative values indicate that cytokine concentration was lower in the exposed group; positive values indicate that cytokine concentration was higher in the exposed group.

^b^A minimal false discovery rate (*q*-value) for significance.

^c^Values are median (25th percentile, 75th percentile). The units of BDNF, ALCAM, and MSP are ng/mL; the unit of CRP is mg/L.

**Table 3:** Associations of creatinine-standardized urinary PAH metabolites with cytokine expression levels (as the dependent variable).

Variable^a^	BDNF^a^		ALCAM^a^		CRP^a^		MSP^a^	
	*β*(95%CI)	*p*^b^	*β*(95%CI)	*p*^b^	*β*(95%CI)	*p*^b^	*β*(95%CI)	*p*^b^
1-hydroxypyrene	−0.135 (−0.256, −0.013)	**0.030**	0.053 (0.008, 0.097)	**0.020**	0.344 (0.218, 0.469)	**<0.001**	0.061 (−0.021, 0.143)	0.143
1-hydroxynaphthalene	−0.065 (−0.194, 0.065)	0.326	0.050 (0.003, 0.097)	**0.037**	0.208 (0.072, 0.344)	**0.003**	0.016 (−0.071, 0.102)	0.723
2-hydroxynaphthalene	−0.047 (−0.183, 0.089)	0.498	0.029 (−0.020, 0.078)	0.248	0.228 (0.085, 0.371)	**0.002**	−0.055 (−0.145, 0.036)	0.238
2-hydroxyfluorene	−0.076 (−0.205, 0.052)	0.244	0.059 (0.013, 0.106)	**0.012**	0.243 (0.108, 0.377)	**<0.001**	−0.005 (−0.091, 0.081)	0.908
9-hydroxyfluorene	−0.062 (−0.111, −0.013)	**0.014**	0.010 (−0.008, 0.028)	0.264	−0.003 (−0.055, 0.050)	0.920	0.004 (−0.030, 0.037)	0.825
1-hydroxyphenanthrene	−0.003 (−0.092, 0.086)	0.953	0.010 (−0.022, 0.043)	0.535	0.149 (0.055, 0.242)	**0.002**	0.039 (−0.021, 0.098)	0.200
2-hydroxyphenanthrene	0.005 (−0.098, 0.109)	0.922	0.064 (0.027, 0.101)	**0.001**	0.200 (0.092, 0.308)	**<0.001**	0.027 (−0.042, 0.097)	0.436
3-hydroxyphenanthrene	0.012 (−0.074, 0.099)	0.781	0.035 (0.004, 0.066)	**0.027**	0.108 (0.017, 0.199)	**0.020**	−0.022 (−0.080, 0.036)	0.450
4-hydroxyphenanthrene	−0.062 (−0.113, −0.011)	**0.018**	0.004 (−0.015, 0.023)	0.676	0.014 (−0.041, 0.068)	0.625	0.003 (−0.032, 0.037)	0.884
9-hydroxyphenanthrene	−0.065 (−0.168, 0.037)	0.212	0.035 (−0.002, 0.072)	0.065	0.155 (0.047, 0.263)	**0.005**	0.061 (−0.008, 0.130)	0.081
ΣOH-PAHs	−0.143 (−0.302, 0.016)	0.078	0.067 (0.009, 0.124)	**0.023**	0.389 (0.224, 0.555)	**<0.001**	0.048 (−0.059, 0.154)	0.380

^a^Parameters were ln-transformed prior to inclusion in the analysis.

^b^Adjusted for age, gender, BMI, smoking status, pack-years of smoking, alcohol use status, working years, and exercise.

**Table 4:** Associations of plasma cytokines with HRV indices (as the dependent variable).

Variable	SDNN^a^		r-MSSD^a^		LF^a^		HF^a^		Tp^a^	
	*β*(95%CI)	*P*	*β*(95%CI)	*P*	*β*(95%CI)	*P*	*β*(95%CI)	*P*	*β*(95%CI)	*P*
**model 1**^b^										
BDNF	−0.018(−0.044, 0.009)	0.184	−0.029(−0.057, −0.001)	**0.042**	−0.042(−0.111, 0.026)	0.227	−0.088(−0.165, −0.011)	**0.025**	−0.035(−0.094, 0.024)	0.240
ALCAM	−0.048(−0.121, 0.025)	0.198	−0.040(−0.118, 0.037)	0.307	−0.138(−0.327, 0.051)	0.153	−0.214(−0.426, −0.002)	**0.048**	−0.073(−0.235, 0.088)	0.373
CRP	−0.038(−0.064, −0.013)	**0.003**	−0.028(−0.055, −0.001)	**0.039**	−0.083(−0.148, −0.019)	**0.012**	−0.061(−0.134, 0.011)	0.099	−0.058(−0.113, −0.003)	**0.038**
**model 2**^c^										
BDNF	−0.015(−0.042, 0.011)	0.258	−0.028(−0.056, 0.001)	0.055	−0.038(−0.107, 0.032)	0.287	−0.079(−0.156, −0.001)	**0.046**	−0.032(−0.092, 0.027)	0.282
ALCAM	−0.041(−0.115, 0.033)	0.277	−0.029(−0.107, 0.050)	0.473	−0.108(−0.300, 0.085)	0.273	−0.183(−0.399, 0.033)	0.096	−0.056(−0.221, 0.109)	0.503
CRP	−0.038(−0.063, −0.013)	**0.003**	−0.028(−0.054, −0.002)	**0.038**	−0.082(−0.147, −0.017)	**0.013**	−0.061(−0.133, 0.012)	0.100	−0.059(−0.114, −0.003)	**0.038**

^a^Parameters were ln-transformed prior to inclusion in the analysis.

^b^Regression coefficients were adjusted for age (continuous), gender (binary), BMI (continuous), smoking status (binary), pack-years of smoking (continuous), alcohol use status (binary), working years (continuous) and exercise(binary).

^c^Further adjusted for the other three cytokines (continuous).
