# Supplementary material for: Exposure to Polycyclic Aromatic Hydrocarbons, Plasma Cytokines, and Heart Rate Variability
Source: Sci Rep. 2016 Jan 13;6:19272. doi: 10.1038/srep19272 (PMC4725366; doi:10.1038/srep19272)
Supplement: Supplementary Information [file srep19272-s1.doc]

**Supplemental Material**

**Exposure to Polycyclic Aromatic Hydrocarbons**, **Plasma Cytokines**, **and Heart Rate Variability**

Binyao Yang^1,2†^, Qifei Deng^3,1†^,Wangzhen Zhang^4^, Yingying Feng^1^, Xiayun Dai^1^, Wei Feng^1^, Xiaosheng He^1^, Suli Huang^1^, Xiao Zhang^1^, Xiaohai Li^1^, Dafeng Lin^1^, Meian He^1^, Huan Guo^1^, Huizhen Sun^1^, Jing Yuan^1^, Jiachun Lu^2^, Frank B. Hu^5,6,7^, Xiaomin Zhang^1*^ and Tangchun Wu^1*^

**Table of Contents**

| **Title** | **Page number** |
| --- | --- |
| Supplemental Material, Table S1. Distribution of PAH metabolites, plasma cytokines and HRV indices in validation population (n = 489^a^) | 3 |
| Supplemental Material, Table S2. Proteins measured with cytokine antibody array (Quantibody® Human Cytokine Antibody Array 6000) | 4 |
| Supplemental Material, Table S3. Partial correlation coefficienta among plasma cytokines. | 18 |
| Supplemental Material, Table S4. Associations between PAHs metabolites levels and HRV indices (as the dependent variable) in the validation stage. | 19 |
| Supplemental Material, Table S5. Associations of urinary PAHs metabolites with cytokines expression levels (as the dependent variable) | 20 |
| Supplemental Material, Table S6. Partial correlation coefficienta among PAHs metabolites and ΣOH-PAHs | 21 |

| Table S1. Distribution of PAH metabolites, plasma cytokines and HRV indices in validation population (n = 489^a^) | | | | | |
| --- | --- | --- | --- | --- | --- |
|
|  |  | **Percentile** | | | |
| **Variable** | **n** | **25th** | **50th** | **75th** | **IQR** |
| **PAH metabolites** (µg/mmol creatine) | | | | | |
| 1-hydroxypyrene | 489 | 1.75 | 3.00 | 5.01 | 3.26 |
| 1-hydroxynaphthalene | 489 | 0.89 | 1.44 | 2.32 | 1.43 |
| 2-hydroxynaphthalene | 489 | 0.79 | 1.40 | 2.29 | 1.50 |
| 2-hydroxyfluorene | 489 | 0.55 | 0.82 | 1.25 | 0.69 |
| 9-hydroxyfluorene | 489 | 0.20 | 0.48 | 1.12 | 0.92 |
| 1-hydroxyphenanthrene | 489 | 0.37 | 0.75 | 1.37 | 1.01 |
| 2-hydroxyphenanthrene | 489 | 0.18 | 0.28 | 0.48 | 0.30 |
| 3-hydroxyphenanthrene | 489 | 0.20 | 0.33 | 0.56 | 0.36 |
| 4-hydroxyphenanthrene | 489 | 0.11 | 0.28 | 0.62 | 0.51 |
| 9-hydroxyphenanthrene | 489 | 0.38 | 0.65 | 1.18 | 0.80 |
| ΣOH-PAHs | 489 | 7.58 | 10.59 | 15.73 | 8.15 |
| **Plasma cytokines** | | | | | |
| BDNF ( ng/ml) | 489 | 1.75 | 4.35 | 9.74 | 8.00 |
| ALCAM ( ng/ml) | 489 | 80.64 | 106.22 | 134.09 | 53.45 |
| CRP (mg/l) | 489 | 0.45 | 1.14 | 2.20 | 1.74 |
| MSP ( ng/ml) | 487 | 56.49 | 93.52 | 153.49 | 96.99 |
| **HRV indices** |  |  |  |  |  |
| SDNN (msec) | 489 | 31.95 | 39.80 | 49.00 | 17.05 |
| r-MSSD (msec) | 489 | 18.45 | 23.20 | 29.15 | 10.70 |
| LF (msec^2^) | 489 | 217.60 | 375.89 | 637.77 | 420.17 |
| HF (msec2) | 489 | 74.41 | 138.44 | 250.32 | 175.91 |
| TP (msec2) | 489 | 675.50 | 1117.65 | 1762.08 | 1086.59 |
| Abbreviations: IQR, interquartile range.  ^a^ 2 men were not measured for MSP. | | | | | |

| **Table S2. Proteins measured with cytokine antibody array (Quantibody® Human Cytokine Antibody Array 6000)** | | | | | | |
| --- | --- | --- | --- | --- | --- | --- |
| **NO** | **Cytokines Name** | **Official symbol** | **Full name** | **Score (d)** | **Fold Change（exposure/controls）** | ***q*-value (%)** |
| 1 | BDNF | BDNF | Brain-derived neurotrophic factor | -0.7727 | 0.2761 | ＜5.0000 |
| 2 | TARC/CCL17 | CCL17 | Chemokine (C-C motif) ligand 17 | -2.7357 | 0.314 | ＜5.0000 |
| 3 | Prolactin/PRL | PRL | Prolactin | -2.0418 | 0.3275 | ＜5.0000 |
| 4 | LOX-1/OLR1 | OLR1 | Oxidized low density lipoprotein (lectin-like) receptor 1 | -2.0102 | 0.3369 | ＜5.0000 |
| 5 | DR6/TNFRSF21 | TNFRSF21 | Tumor necrosis factor receptor superfamily, member 21 | -0.9547 | 0.3553 | ＜5.0000 |
| 6 | PDGF-BB | PDGFB | Platelet-derived growth factor beta polypeptide | -3.3188 | 0.3794 | ＜5.0000 |
| 7 | EGF | EGF | Epidermal growth factor | -0.4145 | 0.3857 | ＜5.0000 |
| 8 | IL-16 | IL16 | Interleukin 16 | -1.9129 | 0.3978 | ＜5.0000 |
| 9 | GM-CSF/CSF2 | CSF2 | Colony stimulating factor 2 (granulocyte-macrophage) | -4.3857 | 0.401 | ＜5.0000 |
| 10 | ENA-78/CXCL5 | CXCL5 | Chemokine (C-X-C motif) ligand 5 | -0.2514 | 0.4222 | ＜5.0000 |
| 11 | IFNγ | IFNG | Interferon gamma | -1.0057 | 0.4596 | ＜5.0000 |
| 12 | PECAM-1 | PECAM1 | Platelet endothelial cell adhesion molecule 1 | -2.2716 | 0.4643 | ＜5.0000 |
| 13 | IL-29 | IFNL1 | Interferon, lambda 1 | -2.5487 | 0.4772 | ＜5.0000 |
| 14 | IL-5 | IL5 | Interleukin 5 | -1.3674 | 0.4959 | ＜5.0000 |
| 15 | CCL28 | CCL28 | Chemokine (C-C motif) ligand 28 | -1.1209 | 0.5211 | ＜5.0000 |
| 16 | ALCAM | ALCAM | Activated leukocyte cell adhesion molecule | 3.95 | 2.7382 | ＜5.0000 |
| 17 | MSP | MST1 | Macrophage stimulating 1 (hepatocyte growth factor-like) | 3.0455 | 2.3049 | ＜5.0000 |
| 18 | CRP | CRP | C-reactive protein, pentraxin-related | 2.842 | 2.0121 | ＜5.0000 |
| 19 | FGF-7 | FGF7 | Fibroblast growth factor 7 | 2.9765 | 1.9613 | ＜5.0000 |
| 20 | LAP/TGFB1 | TGFB1 | Transforming growth factor, beta 1 | -0.8599 | 0.5452 | ＜5.0000 |
| 21 | TECK/CCL25 | CCL25 | Chemokine (C-C motif) ligand 25 | -2.1447 | 0.5518 | ＜5.0000 |
| 22 | MIP-3α/CCL20 | CCL20 | Chemokine (C-C motif) ligand 20 | -0.5499 | 0.5554 | ＜5.0000 |
| 23 | IL-9 | IL9 | Interleukin 9 | -0.9325 | 0.5632 | ＜5.0000 |
| 24 | PDGF-AB | PDGFAB | Platelet-derived growth factor subunit AB | -2.3451 | 0.5682 | ＜5.0000 |
| 25 | Lymphotactin/XCL1 | XCL1 | Chemokine (C motif) ligand 1 | -0.2375 | 0.5695 | ＜5.0000 |
| 26 | PDGF-AA | PDGFA | Platelet-derived growth factor alpha polypeptide | -2.7631 | 0.572 | ＜5.0000 |
| 27 | IL-8 | IL8 | Interleukin 8 | -2.3126 | 0.5729 | ＜5.0000 |
| 28 | Kallikrein14 | KLK14 | Kallikrein-related peptidase 14 | -2.8067 | 0.5769 | ＜5.0000 |
| 29 | IL-7 | IL7 | Interleukin 7 | -1.9145 | 0.5821 | ＜5.0000 |
| 30 | MCP-3/CCL7 | CCL7 | Chemokine (C-C motif) ligand 7 | -2.417 | 0.5896 | ＜5.0000 |
| 31 | MIG/CXCL9 | CXCL9 | Chemokine (C-X-C motif) ligand 9 | -1.513 | 0.6282 | ＜5.0000 |
| 32 | ANG-1 | ANGPT1 | Angiopoietin 1 | -0.573 | 0.634 | ＜5.0000 |
| 33 | MIF | MIF | Macrophage migration inhibitory factor (glycosylation-inhibiting factor) | -3.3346 | 0.653 | ＜5.0000 |
| 34 | ActivinA/INHBA | INHBA | Activin A | -0.7302 | 0.6659 | ＜5.0000 |
| 35 | AGRP | AGRP | Agouti related protein homolog (mouse) | -1.8634 | 0.671 | ＜5.0000 |
| 36 | Angiostatin/PLG | PLG | Plasminogen | -0.9196 | 0.7035 | ＜5.0000 |
| 37 | IP-10/CXCL10 | CXCL10 | Chemokine (C-X-C motif) ligand 10 | -2.6865 | 0.7071 | ＜5.0000 |
| 38 | PAI-1 | SERPINE1 | Serpin peptidase inhibitor, clade E (nexin, plasminogen activator inhibitor type 1), member 1 | -2.24 | 0.7141 | ＜5.0000 |
| 39 | SDF-1β/CXCL12B | CXCL12b | Chemokine (C-X-C motif) ligand 12b (stromal cell-derived factor 1) | -1.8888 | 0.7156 | ＜5.0000 |
| 40 | IL-23 | IL23A | Interleukin 23, alpha subunit p19 | -0.8329 | 0.7245 | ＜5.0000 |
| 41 | Follistatin/FST | FST | Follistatin | -0.5272 | 0.7556 | ＜5.0000 |
| 42 | NAP-2 | NAP1L4 | Nucleosome assembly protein 1-like 4 | -0.7369 | 0.767 | ＜5.0000 |
| 43 | MMP-10 | MMP10 | Matrix metallopeptidase 10 (stromelysin 2) | 0.6056 | 20.375 | 28.9286 |
| 44 | IGF-1SR | IGF1R | Insulin-like growth factor 1 receptor | 0.4325 | 9.7736 | 28.9286 |
| 45 | IL-21R | IL21R | Interleukin 21 receptor | 2.5932 | 6.6825 | 10.2101 |
| 46 | Thyroglobulin | TG | Thyroglobulin | 0.5425 | 5.9282 | 28.9286 |
| 47 | IGF-II | IGF2 | Insulin-like growth factor 2 (somatomedin A) | 0.5319 | 5.445 | 28.9286 |
| 48 | XEDAR | EDA2R | Ectodysplasin A2 receptor | -0.8022 | 3.7465 | 15.9606 |
| 49 | NRG1-β1 | NRG1 | Neuregulin 1 | 1.8025 | 3.5687 | 18.8474 |
| 50 | IL-4 | IL4 | Interleukin 4 | -2.1868 | 3.3236 | 18.0804 |
| 51 | IL-2Rγ | IL2RG | Interleukin 2 receptor, gamma | 1.5129 | 2.9846 | 19.8614 |
| 52 | MMP-13 | MMP13 | Matrix metallopeptidase 13 (collagenase 3) | 0.1229 | 2.8988 | 28.9286 |
| 53 | VEGFR3/FLT4 | FLT4 | Fms-related tyrosine kinase 4 | 0.7661 | 2.5796 | 28.9286 |
| 54 | IL-18Rβ | IL18RAP | Interleukin-18 receptor accessory protein | 0.1214 | 2.5234 | 28.9286 |
| 55 | I-309/CCL1 | CCL1 | Chemokine (C-C motif) ligand 1 | 2.083 | 2.4166 | 17.8022 |
| 56 | MMP-2 | MMP2 | Matrix metallopeptidase 2 (gelatinase A, 72kda gelatinase, 72kda type IV collagenase) | 2.1341 | 1.9241 | 17.8022 |
| 57 | MICA | MICA | MHC class I polypeptide-related sequence A | 1.1744 | 1.8318 | 19.8614 |
| 58 | PIGF | PIGF | Phosphatidylinositol glycan anchor biosynthesis, class F | 1.6553 | 1.7536 | 19.8614 |
| 59 | B7-1/CD80 | CD80 | CD80 molecule | 1.2077 | 1.7472 | 19.8614 |
| 60 | Procalcitonin/CALCA | CALCA | Calcitonin-related polypeptide alpha | 1.226 | 1.7189 | 19.8614 |
| 61 | BCAM | BCAM | Basal cell adhesion molecule (Lutheran blood group) | 1.8697 | 1.6736 | 18.8474 |
| 62 | TRAILR3 | TNFRSF10C | Tumor necrosis factor receptor superfamily, member 10c, decoy without an intracellular domain | 2.2387 | 1.6293 | 17.8022 |
| 63 | TGFβ3 | TGFB3 | Transforming growth factor, beta 3 | 0.7883 | 1.5934 | 28.9286 |
| 64 | IGF-I | IGF1 | Insulin-like growth factor 1 (somatomedin C) | 0.8966 | 1.5724 | 28.9286 |
| 65 | GH | GH1 | Growth hormone 1 | 1.3477 | 1.528 | 19.8614 |
| 66 | IL-12p70 | IL12A | Interleukin 12A (natural killer cell stimulatory factor 1, cytotoxic lymphocyte maturation factor 1, p35) | 0.7899 | 1.4488 | 28.9286 |
| 67 | CEACAM-1 | CEACAM1 | Carcinoembryonic antigen-related cell adhesion molecule 1 | 1.3533 | 1.4004 | 19.8614 |
| 68 | TNFsRI | TNFRSF1A | Tumor necrosis factor receptor superfamily, member 1A | 1.3617 | 1.365 | 19.8614 |
| 69 | FSH | FSH | Follicle-stimulating hormone | 0.7999 | 1.3631 | 28.9286 |
| 70 | IL-10Rβ | IL10RB | Interleukin 10 receptor, beta | 0.8958 | 1.3484 | 28.9286 |
| 71 | MPIF-1/CCL23 | CCL23 | Chemokine (C-C motif) ligand 23 | 1.457 | 1.3468 | 19.8614 |
| 72 | CEA | CEACAM5 | Carcinoembryonic antigen-related cell adhesion molecule 5 | 0.133 | 1.3249 | 28.9286 |
| 73 | OPN/SPP1 | SPP1 | Secreted phosphoprotein 1 | 0.5425 | 1.323 | 28.9286 |
| 74 | CD97 | CD97 | CD97 molecule | 1.6082 | 1.3224 | 19.8614 |
| 75 | TNFsRII | TNFRSF1B | Tumor necrosis factor receptor superfamily, member 1B | 1.4186 | 1.322 | 19.8614 |
| 76 | IFNα/βR2 | IFNAR2 | Interferon (alpha, beta and omega) receptor 2 | 1.2103 | 1.3141 | 19.8614 |
| 77 | IL-1RI | IL1R1 | Interleukin-1 receptor type I | 0.9188 | 1.2997 | 28.9286 |
| 78 | uPAR | PLAUR | Plasminogen activator, urokinase receptor | 1.5908 | 1.2962 | 19.8614 |
| 79 | NT-4 | NTF4 | Neurotrophin 4 | 1.3037 | 1.2935 | 19.8614 |
| 80 | DAN/NBL1 | NBL1 | Neuroblastoma 1, DAN family BMP antagonist | 0.7636 | 1.2579 | 28.9286 |
| 81 | IL-1R4/ST2 | ST2 | Suppression of tumorigenicity 2 | 0.8569 | 1.2579 | 28.9286 |
| 82 | HVEM/TNFRSF14 | TNFRSF14 | Tumor necrosis factor receptor superfamily, member 14 | 0.7711 | 1.2556 | 28.9286 |
| 83 | VCAM-1 | VCAM1 | Vascular cell adhesion molecule 1 | 1.6071 | 1.2541 | 19.8614 |
| 84 | IL-17R | IL17RA | Interleukin 17 receptor A | 1.0578 | 1.2516 | 28.9286 |
| 85 | SCF/KITLG | KITLG | KIT ligand | 0.897 | 1.2508 | 28.9286 |
| 86 | hemerin/RARRES2 | RARRES2 | Retinoic acid receptor responder (tazarotene induced) 2 | 1.1631 | 1.2425 | 19.8614 |
| 87 | Dtk/TYRO3 | TYRO3 | TYRO3 protein tyrosine kinase | 1.2271 | 1.2229 | 19.8614 |
| 88 | PARC/CCL18 | CCL18 | Chemokine (C-C motif) ligand 18 (pulmonary and activation-regulated) | 1.9417 | 1.2151 | 18.8474 |
| 89 | IL-1sRII | IL1R2 | Interleukin-1 receptor type II | -0.6469 | 1.2126 | 10.2101 |
| 90 | TGFβ1 | TGFB1 | Transforming growth factor, beta 1 | 1.2786 | 1.2081 | 19.8614 |
| 91 | PF4 | PF4 | Platelet factor 4 | 1.0775 | 1.1793 | 28.9286 |
| 92 | bFGF | FGF2 | Fibroblast growth factor 2 (basic) | 0.4543 | 1.1773 | 28.9286 |
| 93 | ErbB3 | ERBB3 | V-erb-b2 avian erythroblastic leukemia viral oncogene homolog 3 | 0.7978 | 1.1609 | 28.9286 |
| 94 | CD14 | CD14 | CD14 molecule | 1.3232 | 1.1604 | 19.8614 |
| 95 | BMP-5 | BMP5 | Bone morphogenetic protein 5 | 2.6514 | 1.1555 | 10.2101 |
| 96 | HCC-1 | CCL14 | Chemokine (C-C motif) ligand 14 | 0.9433 | 1.1499 | 28.9286 |
| 97 | RAGE/AGER | AGER | Advanced glycosylation end product-specific receptor | 0.8664 | 1.1479 | 28.9286 |
| 98 | NT-3 | NTF3 | Neurotrophin 3 | 0.0906 | 1.1456 | 28.9286 |
| 99 | VEGFR2 | KDR | Kinase insert domain receptor (a type III receptor tyrosine kinase) | 0.6104 | 1.1439 | 28.9286 |
| 100 | CD200 | CD200 | CD200 molecule | 0.5704 | 1.1412 | 28.9286 |
| 101 | BCMA/TNFRSF17 | TNFRSF17 | Tumor necrosis factor receptor superfamily, member 17 | 0.9149 | 1.1335 | 28.9286 |
| 102 | Adipsin | Adipsin | Adipsin | 0.6841 | 1.133 | 28.9286 |
| 103 | L-Selectin/SELL | Sell | Selectin, lymphocyte | 0.7355 | 1.1047 | 28.9286 |
| 104 | IGFBP-3 | IGFBP3 | Insulin-like growth factor binding protein 3 | 0.2447 | 1.1005 | 28.9286 |
| 105 | MICB | MICB | MHC class I polypeptide-related sequence B | 0.9357 | 1.0964 | 28.9286 |
| 106 | TIMP-1 | TIMP1 | TIMP metallopeptidase inhibitor 1 | 0.6323 | 1.0955 | 28.9286 |
| 107 | NCAM-1 | NCAM1 | Neural cell adhesion molecule 1 | 0.1966 | 1.0863 | 28.9286 |
| 108 | GDF-15 | GDF15 | Growth differentiation factor 15 | 0.4017 | 1.0796 | 28.9286 |
| 109 | Eotaxin/CCL11 | CCL11 | Chemokine (C-C motif) ligand 11 | 0.6036 | 1.0676 | 28.9286 |
| 110 | LYVE-1 | LYVE1 | Lymphatic vessel endothelial hyaluronic acid receptor 1 | 0.6557 | 1.0601 | 28.9286 |
| 111 | Ferritin | FTL | Ferritin, light polypeptide | 0.6429 | 1.0533 | 28.9286 |
| 112 | Notch-1 | NOTCH1 | Notch 1 | 0.4122 | 1.0477 | 28.9286 |
| 113 | Trappin-2/PI3 | PI3 | Peptidase inhibitor 3, skin-derived | 0.5028 | 1.0456 | 28.9286 |
| 114 | Endoglin | ENG | Endoglin | 0.3301 | 1.0447 | 28.9286 |
| 115 | HCC-4/CCL16 | CCL16 | Chemokine (C-C motif) ligand 16 | 0.5851 | 1.0384 | 28.9286 |
| 116 | ICAM-1 | ICAM1 | Intercellular adhesion molecule 1 | 0.5094 | 1.0372 | 28.9286 |
| 117 | IGFBP-2 | IGFBP2 | Insulin-like growth factor binding protein 2 | 0.3321 | 1.0311 | 28.9286 |
| 118 | SCFR/KIT | KIT | V-kit Hardy-Zuckerman 4 feline sarcoma viral oncogene homolog | 0.386 | 1.0306 | 28.9286 |
| 119 | Siglec-5 | SIGLEC5 | Sialic acid binding Ig-like lectin 5 | -0.6452 | 1.0037 | 17.449 |
| 120 | Angiogenin | ANG | Angiogenin, ribonuclease, rnase A family, 5 | 0.0077 | 1.0025 | 28.9286 |
| 121 | IL-28A | IFNL2 | Interferon, lambda 2 | 0 | 1 | 28.9286 |
| 122 | OSM | OSM | Oncostatin M | 0 | 1 | 28.9286 |
| 123 | TACE/ADAM17 | ADAM17 | ADAM metallopeptidase domain 17 | 0 | 1 | 28.9286 |
| 124 | BMP-7 | BMP 7 | Bone morphogenetic protein 7 | 1.5497 | 0.9992 | 19.8614 |
| 125 | ICAM-2 | ICAM2 | Intercellular adhesion molecule 2 | -2.441 | 0.9972 | 18.0804 |
| 126 | IGFBP-6 | IGFBP6 | Insulin-like growth factor binding protein 6 | -1.1108 | 0.9913 | 18.0804 |
| 127 | HGFR/MET | MET | Met proto-oncogene | -0.1222 | 0.9884 | 18.0804 |
| 128 | BLC | CXCL13 | Chemokine (C-X-C motif) ligand 13 | 0.1615 | 0.9849 | 28.9286 |
| 129 | MCSFR | CSF1R | Colony stimulating factor 1 receptor | -1.8658 | 0.9844 | 18.0804 |
| 130 | FAP | FAP | Fibroblast activation protein, alpha | -0.362 | 0.9829 | 18.0804 |
| 131 | SerpinA4 | SERPINA4 | Serpin peptidase inhibitor, clade A (alpha-1 antiproteinase, antitrypsin), member 4 | -0.2171 | 0.9829 | 18.0804 |
| 132 | MBL | MBL2 | Mannose-binding lectin (protein C) 2, soluble | -0.1026 | 0.9804 | 18.0804 |
| 133 | IL-6sR | IL6R | Interleukin 6 receptor | -2.0124 | 0.9798 | 18.0804 |
| 134 | PGRP-5 | pglyrp5 | Peptidoglycan recognition proteins 5 | -0.2436 | 0.9796 | 18.0804 |
| 135 | RANTES/CCL5 | CCL5 | C-C motif chemokine 5 | -0.1834 | 0.9779 | 18.0804 |
| 136 | AR | AR | Androgen receptor | 1.2269 | 0.9616 | 19.8614 |
| 137 | Adiponectin | ADIPOQ | Adiponectin, C1Q and collagen domain containing | -0.6388 | 0.9556 | 18.0804 |
| 138 | Eotaxin-2/CCL24 | CCL24 | Chemokine (C-C motif) ligand 24 | -0.2868 | 0.9544 | 18.0804 |
| 139 | sgp130/IL6ST | IL6ST | Interleukin 6 signal transducer (gp130, oncostatin M receptor) | -0.4803 | 0.9536 | 18.0804 |
| 140 | CTACK | CCL27 | Chemokine (C-C motif) ligand 27 | -0.6928 | 0.9502 | 18.0804 |
| 141 | NrCAM | NRCAM | Neuronal cell adhesion molecule | -0.2596 | 0.9478 | 18.0804 |
| 142 | Lipocalin-2 | LCN2 | Lipocalin 2 | -2.1562 | 0.9425 | 18.0804 |
| 143 | ANG-2 | ANGPT2 | Angiopoietin 2 | -1.9737 | 0.9313 | 18.0804 |
| 144 | NOV | NOV | Nephroblastoma overexpressed | -0.6336 | 0.9305 | 17.449 |
| 145 | LGMN | LGMN | Legumain | -0.6644 | 0.9236 | 18.0804 |
| 146 | TIMP-4 | TIMP4 | TIMP metallopeptidase inhibitor 4 | -0.2263 | 0.9202 | 18.0804 |
| 147 | EG-VEGF/PROK1 | PROK1 | Prokineticin 1 Growth Factor | 0.5681 | 0.9104 | 28.9286 |
| 148 | FGF-4 | FGF4 | Fibroblast growth factor 4 | 0.5065 | 0.9054 | 28.9286 |
| 149 | DcR3/TNFRSF6B | TNFRSF6B | Tumor necrosis factor receptor superfamily, member 6b, decoy | -1.1462 | 0.9013 | 15.9606 |
| 150 | THBD | THBD | Thrombomodulin | -0.334 | 0.8978 | 18.0804 |
| 151 | IL-1RL1 | IL1RL1 | Interleukin 1 receptor-like 1 | -1.0859 | 0.8974 | 10.2101 |
| 152 | HGF | HGF | Hepatocyte growth factor (hepapoietin A; scatter factor) | -0.1209 | 0.8969 | 18.0804 |
| 153 | EpCAM | EPCAM | Epithelial cell adhesion molecule | 0.0478 | 0.889 | 28.9286 |
| 154 | IL-13R2 | IL13RA2 | Interleukin 13 receptor, alpha 2 | -5.6321 | 0.8879 | 10.2101 |
| 155 | Nidogen-1 | NID1 | Nidogen 1 | -1.263 | 0.8856 | 8.617 |
| 156 | IL-2Rα | IL2RA | Interleukin 2 receptor, alpha | -1.2842 | 0.881 | 10.2101 |
| 157 | VEGF-C | VEGFC | Vascular endothelial growth factor C | -1.0387 | 0.8762 | 10.2101 |
| 158 | Flt-3L | FLT3LG | Fms-like tyrosine kinase 3 Ligand | -1.6341 | 0.8744 | 18.0804 |
| 159 | E-Cadherin/CDH1 | CDH1 | Cadherin 1, type 1, E-cadherin (epithelial) | -4.0407 | 0.8739 | 14.7273 |
| 160 | Lep | LEP | Leptin | -0.0082 | 0.869 | 15.9606 |
| 161 | BMP-2 | BMP2 | Bone morphogenetic protein 2 | -0.3024 | 0.8635 | 15.9606 |
| 162 | TGF-β2 | TGFB2 | Transforming growth factor, beta 2 | -1.2349 | 0.861 | 8.617 |
| 163 | Galectin-3 | LGALS3 | Lectin, galactoside-binding, soluble, 3 | -0.6454 | 0.8556 | 18.0804 |
| 164 | GITR/TNFRSF18 | TNFRSF18 | Tumor necrosis factor receptor superfamily, member 18 | 1.0253 | 0.8545 | 28.9286 |
| 165 | IL-12p40 | IL12B | Interleukin 12B (natural killer cell stimulatory factor 2, cytotoxic lymphocyte maturation factor 2, p40) | -2.0729 | 0.8536 | 7.5466 |
| 166 | IL-17B | IL17B | Interleukin 17B | -1.3095 | 0.8526 | 16.6707 |
| 167 | Resistin/RETN | RETN | Resistin | -1.1551 | 0.8518 | 10.2101 |
| 168 | DKK-1 | DKK1 | Dickkopf WNT signaling pathway inhibitor 1 | -2.3572 | 0.8511 | 10.2101 |
| 169 | IL-24 | IL24 | Interleukin 24 | -2.4015 | 0.8457 | 15.9606 |
| 170 | AXL/UFO | AXL | AXL receptor tyrosine kinase | -0.5256 | 0.8427 | 14.7273 |
| 171 | IL-2Rβ | IL2RB | Interleukin 2 receptor, beta | -0.3581 | 0.8423 | 8.617 |
| 172 | TPO | TPO | Thyroid peroxidase | -1.3451 | 0.8422 | 7.5466 |
| 173 | Transferrin/TF | TF | Transferrin | -0.8329 | 0.8393 | 15.9606 |
| 174 | FasL | FASLG | Fas ligand (TNF superfamily, member 6) | -1.4946 | 0.8331 | 7.5466 |
| 175 | BMP-4 | BMP4 | Bone morphogenetic protein 4 | -1.0298 | 0.8316 | 18.0804 |
| 176 | TNFα | TNF | Tumor necrosis factor | 0.4025 | 0.8297 | 28.9286 |
| 177 | MIP-1β/CCL4 | CCL4 | Chemokine (C-C motif) ligand 4 | -1.5411 | 0.8222 | 6.5747 |
| 178 | IGFBP-4 | IGFBP4 | Insulin-like growth factor binding protein 4 | 0.7153 | 0.8206 | 28.9286 |
| 179 | TREM-1 | TREM1 | Triggering receptor expressed on myeloid cells 1 | -1.4403 | 0.8198 | 7.5466 |
| 180 | CatheprinS/CTSS | CTSS | Cathepsin S | -0.8313 | 0.8173 | 10.2101 |
| 181 | Cripto-1/TDGF1 | TDGF1 | Teratocarcinoma-derived growth factor 1 | -0.0631 | 0.8151 | 6.5747 |
| 182 | GDNF | GDNF | Glial cell derived neurotrophic factor | 0.1929 | 0.8119 | 28.9286 |
| 183 | E-Selectin/SELE | SELE | Selectin E | -0.5098 | 0.8114 | 14.7273 |
| 184 | WIF-1 | WIF1 | Wnt inhibitory factor 1 | -0.5058 | 0.8092 | 18.0804 |
| 185 | TRAILR4 | TNFRSF10D | Tumor necrosis factor receptor superfamily, member 10d, decoy with truncated death domain | -1.3388 | 0.8007 | 8.617 |
| 186 | ShhN | Shh N | Sonic hedgehog n-terminus | -1.5607 | 0.8003 | 6.5747 |
| 187 | FcγRIIB/C | FCGR2B | Fc fragment of igg, low affinity iib, receptor (CD32) | -0.4791 | 0.7939 | 6.5747 |
| 188 | MCP-1 | CCL2 | Chemokine (C-C motif) ligand 2 | 0.8269 | 0.7878 | 28.9286 |
| 189 | MMP-3 | MMP3 | Matrix metallopeptidase 3 (stromelysin 1, progelatinase) | -1.9856 | 0.7863 | 15.9606 |
| 190 | BMP-9/GDF2 | GDF2 | Growth differentiation factor 2 | -0.8577 | 0.7838 | 10.2101 |
| 191 | CD40 | CD40 | CD40 molecule, TNF receptor superfamily member 5 | -0.8093 | 0.7836 | 10.2101 |
| 192 | IL-13R1 | IL13RA1 | Interleukin 13 receptor, alpha 1 | -1.0964 | 0.7807 | 6.5747 |
| 193 | ANGPTL4 | ANGPTL4 | Angiopoietin-like 4 | 0.8492 | 0.7801 | 28.9286 |
| 194 | B2M | B2M | Beta-2-microglobulin | -2.3138 | 0.7778 | 18.0804 |
| 195 | OPG/TNFRSF11B | TNFRSF11B | Tumor necrosis factor receptor superfamily, member 11b | -0.5986 | 0.7773 | 18.0804 |
| 196 | VEGF-D/FIGF | FIGF | C-fos induced growth factor (vascular endothelial growth factor D) | 0.2506 | 0.7769 | 28.9286 |
| 197 | IGF-IIR | IGF2R | Insulin-like growth factor II receptor | -1.3225 | 0.7761 | 10.2101 |
| 198 | APRIL | TNFSF13 | Tumor necrosis factor (ligand) superfamily, member 13 | 0.1776 | 0.7727 | 28.9286 |
| 199 | VEGFR1/FLT1 | FLT1 | Fms-related tyrosine kinase 1 | -1.3559 | 0.7712 | 7.5466 |
| 200 | Tie-2/TEK | TEK | TEK tyrosine kinase, endothelial | -1.4788 | 0.7601 | 6.5747 |
| 201 | TIMP-2 | TIMP2 | TIMP metallopeptidase inhibitor 2 | -0.8208 | 0.7586 | 15.9606 |
| 202 | FGF-19 | FGF19 | Fibroblast growth factor 19 | 0.1086 | 0.7545 | 28.9286 |
| 203 | MMP-9 | MMP9 | Matrix metallopeptidase 9 (gelatinase B, 92kda gelatinase, 92kda type IV collagenase) | -1.9594 | 0.7424 | 14.7273 |
| 204 | Insulin | INS | Insulin | -2.0413 | 0.7343 | 14.7273 |
| 205 | CD40L | CD40LG | Cd40 ligand | -1.4523 | 0.7224 | 16.6707 |
| 206 | MIP-1δ/CCL15 | CCL15 | Chemokine (C-C motif) ligand 15 | -2.5134 | 0.7158 | 6.5747 |
| 207 | TNFβ | LTA | Lymphotoxin alpha | -1.8075 | 0.6998 | 8.1274 |
| 208 | Osteoactivin | GPNMB | Glycoprotein (transmembrane) nmb | -0.9443 | 0.6831 | 14.7273 |
| 209 | CXCL16 | CXCL16 | Chemokine (C-X-C motif) ligand 16 | -0.8593 | 0.677 | 16.6707 |
| 210 | β-NGF | NGF | Nerve growth factor (beta polypeptide) | -0.8679 | 0.676 | 14.7273 |
| 211 | IL-10 | IL10 | Interleukin 10 | -1.1905 | 0.6702 | 17.449 |
| 212 | sFRP-3/FRZB | FRZB | Frizzled-related protein | 0.3628 | 0.6685 | 28.9286 |
| 213 | FABP2 | FABP2 | Fatty acid binding protein 2, intestinal | -0.1965 | 0.6573 | 18.0804 |
| 214 | AFP | AFP | Alpha-1-fetoprotein | -1.9813 | 0.6291 | 17.449 |
| 215 | IL-1R6 | IL1RL2 | Interleukin 1 receptor-like 2 | -1.2101 | 0.6285 | 8.617 |
| 216 | Eotaxin-3/CCL26 | CCL26 | Chemokine (C-C motif) ligand 26 | -0.9743 | 0.6183 | 18.0804 |
| 217 | IGFBP-1 | IGFBP1 | Insulin-like growth factor binding protein 1 | -0.053 | 0.6159 | 10.2101 |
| 218 | G-CSF | CSF3 | Colony stimulating factor 3 (granulocyte) | -2.4054 | 0.6077 | 18.0804 |
| 219 | IL-6 | IL6 | Interleukin 6 (interferon, beta 2) | -0.2175 | 0.6062 | 7.5466 |
| 220 | IL-3 | IL3 | Interleukin 3 (colony-stimulating factor, multiple) | -0.5377 | 0.5896 | 12.245 |
| 221 | EGFR | EGFR | Epidermal growth factor receptor | -3.7662 | 0.5833 | 18.0804 |
| 222 | 4-1BB/TNFRSF9 | TNFRSF9 | Tumor necrosis factor receptor superfamily member 9 | 0.0517 | 0.5766 | 28.9286 |
| 223 | ErbB2 | ERBB2 | V-erb-b2 avian erythroblastic leukemia viral oncogene homolog 2 | 0.3343 | 0.5728 | 28.9286 |
| 224 | Neprilysin/MME | MME | Membrane metallo-endopeptidase | -0.7369 | 0.5696 | 16.6707 |
| 225 | IL-21 | IL21 | Interleukin 21 | -1.6081 | 0.5529 | 18.0804 |
| 226 | GRO/CXCR2 | CXCR2 | Chemokine (C-X-C motif) receptor 2 | -2.4709 | 0.5446 | 5.3047 |
| 227 | PDGFRβ | PDGFRB | Platelet-derived growth factor receptor, beta polypeptide | -1.0662 | 0.5421 | 10.2101 |
| 228 | C5a | C5 | Complement component 5a | -0.9694 | 0.5297 | 7.5466 |
| 229 | MCP-4 | CCL13 | Chemokine (C-C motif) ligand 13 | -2.9741 | 0.5294 | 5.4107 |
| 230 | IL-1αa | IL1A | Interleukin 1, alpha | -0.4516 | 0.5214 | 10.2101 |
| 231 | TSLP | TSLP | Thymic stromal lymphopoietin | -1.9324 | 0.5101 | 6.1274 |
| 232 | PSA/KLK3 | KLK3 | Kallikrein-related peptidase 3 | 0.1683 | 0.5083 | 28.9286 |
| 233 | NGFR | NGFR | Nerve growth factor receptor | -0.9202 | 0.5068 | 14.7273 |
| 234 | VEGF | VEGFA | Vascular endothelial growth factor A | -0.5591 | 0.5 | 18.0804 |
| 235 | CathepsinL/CTSL | CTSL | Cathepsin L | -2.6332 | 0.4976 | 15.9606 |
| 236 | TRAILR1 | TNFRSF10A | Tumor necrosis factor receptor superfamily, member 10a | -0.6648 | 0.4941 | 17.449 |
| 237 | CD30/TNFRSF8 | TNFRSF8 | Tumor necrosis factor receptor superfamily, member 8 | -0.731 | 0.4912 | 15.9606 |
| 238 | IL-13 | IL13 | Interleukin 13 | -1.5758 | 0.4838 | 8.1274 |
| 239 | MCP-2/CCL8 | CCL8 | Chemokine (C-C motif) ligand 8 | -2.0292 | 0.4389 | 6.1274 |
| 240 | TLR2 | TLR2 | Toll-like receptor 2 | -0.6062 | 0.4259 | 18.0804 |
| 241 | IL-2 | IL2 | Interleukin 2 | -1.0004 | 0.4206 | 10.2101 |
| 242 | Galectin-7/LGALS7 | LGALS7 | Lectin, galactoside-binding, soluble, 7 | -4.2218 | 0.4189 | 17.449 |
| 243 | MMP-1 | MMP1 | Matrix metallopeptidase 1 (interstitial collagenase) | -0.8202 | 0.4069 | 14.7273 |
| 244 | TSH/CGA | CGA | Glycoprotein hormones, alpha polypeptide | -2.0485 | 0.402 | 6.1274 |
| 245 | HB-EGF | HBEGF | Heparin-binding EGF-like growth factor | -2.5621 | 0.3876 | 15.1274 |
| 246 | LIMPII/SCARB2 | SCARB2 | Scavenger receptor class B, member 2 | 0.2372 | 0.3832 | 28.9286 |
| 247 | IL-17 | IL17A | Interleukin 17A | -0.7466 | 0.3631 | 8.1274 |
| 248 | IL-11 | IL11 | Interleukin 11 | -1.3886 | 0.3626 | 10.2101 |
| 249 | I-TAC/CXCL11 | CXCL11 | Chemokine (C-X-C motif) ligand 11 | -1.6812 | 0.3591 | 7.0361 |
| 250 | 2B4/Cd244 | Cd244 | CD244 natural killer cell receptor 2B4 | -0.2415 | 0.3525 | 6.5747 |
| 251 | ADAM9 | ADAM9 | AADAM metallopeptidase domain 9 | -0.4436 | 0.3524 | 16.6707 |
| 252 | HCGβ | CGB | Chorionic gonadotropin, beta polypeptide | -0.1893 | 0.3432 | 17.0476 |
| 253 | SDF-1α/CXCL12 | CXCL12 | Chemokine (C-X-C motif) ligand 12 | 0.4393 | 0.3382 | 28.9286 |
| 254 | MDC | CCL22 | Chemokine (C-C motif) ligand 22 | -1.6465 | 0.3318 | 18.0804 |
| 255 | BTC | BTC | Betacellulin | -1.3679 | 0.3284 | 15.9606 |
| 256 | IL-15 | IL15 | Interleukin 15 | -2.6566 | 0.3277 | 8.032 |
| 257 | TIM-1/HAVCR1 | HAVCR1 | Hepatitis A virus cellular receptor 1 | -1.1415 | 0.3196 | 10.2101 |
| 258 | ICAM-3 | ICAM3 | Intercellular adhesion molecule 3 | -2.178 | 0.315 | 6.4107 |
| 259 | IL-1β | IL1B | Interleukin 1, beta | -1.1979 | 0.3149 | 18.0804 |
| 260 | IL-1ra | IL1RN | Interleukin 1 receptor antagonist | -1.2015 | 0.3138 | 7.5466 |
| 261 | IL-33 | IL33 | Interleukin 33 | -0.2929 | 0.3037 | 10.2101 |
| 262 | MCSF | CSF1 | Colony stimulating factor 1 (macrophage) | -0.3093 | 0.3001 | 19.3569 |
| 263 | MIP-3β/CCL19 | CCL19 | Chemokine (C-C motif) ligand 19 | -0.9223 | 0.2913 | 18.0804 |
| 264 | GCP-2/CXCL6 | CXCL6 | Chemokine (C-X-C motif) ligand 6 | -0.4016 | 0.2832 | 9.0372 |
| 265 | NSE | ENO2 | Enolase 2 (gamma, neuronal) | -5.0452 | 0.2684 | 6.0329 |
| 266 | GROα/CXCL1 | CXCL1 | Chemokine (C-X-C motif) ligand 1 (melanoma growth stimulating activity, alpha) | -2.0332 | 0.2472 | 8.4107 |
| 267 | Siglec-9 | SIGLEC9 | Sialic acid-binding Ig-like lectin 9 | -0.7044 | 0.2246 | 16.6707 |
| 268 | 6Ckine/CCL21 | CCL21 | Chemokine (C-C motif) ligand 21 | -1.7767 | 0.2153 | 18.0804 |
| 269 | PD-1/PDCD1 | PDCD1 | Programmed cell death 1 | -1.0773 | 0.2146 | 10.2101 |
| 270 | MMP-8 | MMP8 | Matrix metallopeptidase 8 (neutrophil collagenase) | -0.869 | 0.2145 | 7.1274 |
| 271 | IL-31 | IL31 | Interleukin 31 | -1.0974 | 0.1953 | 18.0804 |
| 272 | IL-17F | IL17F | Interleukin 17F | -1.3704 | 0.1933 | 8.617 |
| 273 | Fas | FAS | Fas cell surface death receptor | -1.4352 | 0.1224 | 18.0804 |
| 274 | LIGHT/TNFSF14 | TNFSF14 | Tumor necrosis factor (ligand) superfamily, member 14 | -0.5561 | 0.111 | 18.0804 |
| 275 | MIP-1α/CCL3 | CCL3 | Chemokine (C-C motif) ligand 3 | -1.4759 | 0.1001 | 6.5747 |
| 276 | TGFα | TGFA | Transforming growth factor, alpha | NA | NA | NA |
| 277 | LIF | LIF | Leukemia inhibitory factor | NA | NA | NA |
| 278 | CA125 | MUC16 | Mucin 16, cell surface associated | NA | NA | NA |
| 279 | CA15-3 | MUC1 | Mucin 1, cell surface associated | NA | NA | NA |
| 280 | IL-18BPa | IL18BP | Interleukin-18-binding protein | NA | NA | NA |
| Proteins with changes in expression called significant by Significance analysis of microarrays (SAM). Na, Not available. | | | | | | |

| **Table S3. Partial correlation coefficient^a^ among plasma cytokines** | | | | |
| --- | --- | --- | --- | --- |
| **Variable^b^** | **BDNF** | **ALCAM** | **CRP** | **MSP** |
| **BDNF** | 1.000 | 0.075 | 0.062 | -0.096 |
| **ALCAM** | 0.075 | 1.000 | 0.003 | 0.136 |
| **CRP** | 0.062 | 0.003 | 1.000 | 0.093 |
| **MSP** | -0.096 | 0.136 | 0.093 | 1.000 |
| ^a^ Pearson partial correlation with adjustment for Regression coefficients were adjusted for age, gender, BMI, smoking status, pack-years of smoking, alcohol use status, working years, and exercise; ^b^ln-transformed prior to inclusion in the analysis. | | | | |
|
|

| **Table S4. Associations between PAH metabolites levels and HRV indices (as the dependent variable) in the validation stage.** | | | | | | | | | | | | | |  | |
| --- | --- | --- | --- | --- | --- | --- | --- | --- | --- | --- | --- | --- | --- | --- | --- |
| **Variable^a^** | **SDNN** | |  | **r-MSSD** | |  | **LF** | |  | **HF** | |  | **TP** | | |
|  | **β (95% CI)** | ***P^b^*** |  | **β (95% CI)** | ***P^b^*** |  | **β (95% CI)** | ***P^b^*** |  | **β (95% CI)** | ***P^b^*** |  | **β (95% CI)** | | ***P^b^*** |
| 1-hydroxypyrene | 0.017(-0.019, 0.052) | 0.358 |  | 0.009(-0.029, 0.047) | 0.640 |  | -0.006(-0.099, 0.087) | 0.899 |  | 0.028(-0.076, 0.132) | 0.600 |  | 0.038(-0.041, 0.117) | | 0.345 |
| 1-hydroxynaphthalene | -0.003(-0.041, 0.035) | 0.876 |  | -0.002(-0.042, 0.039) | 0.941 |  | -0.097(-0.195, 0.001) | **0.052** |  | -0.045(-0.156, 0.065) | 0.420 |  | -0.039(-0.123, 0.045) | | 0.358 |
| 2-hydroxynaphthalene | -0.020(-0.060, 0.020) | 0.321 |  | -0.024(-0.067, 0.018) | 0.255 |  | -0.083(-0.186, 0.020) | 0.115 |  | -0.090(-0.206, 0.025) | 0.126 |  | -0.035(-0.123, 0.053) | | 0.437 |
| 2-hydroxyfluorene | 0.003(-0.035, 0.041) | 0.881 |  | 0.010(-0.030, 0.050) | 0.611 |  | -0.070(-0.167, 0.028) | 0.162 |  | -0.002(-0.112, 0.108) | 0.971 |  | -0.026(-0.110, 0.057) | | 0.537 |
| 9-hydroxyfluorene | 0.008(-0.006, 0.023) | 0.271 |  | 0.012(-0.004, 0.027) | 0.139 |  | -0.007(-0.045, 0.030) | 0.698 |  | 0.018(-0.024, 0.060) | 0.407 |  | 0.008(-0.025, 0.040) | | 0.645 |
| 1-hydroxyphenanthrene | 0.016(-0.010, 0.042) | 0.231 |  | 0.004(-0.024, 0.032) | 0.775 |  | 0.012(-0.056, 0.080) | 0.728 |  | -0.018(-0.094, 0.058) | 0.644 |  | 0.028(-0.030, 0.085) | | 0.348 |
| 2-hydroxyphenanthrene | -0.006(-0.037, 0.024) | 0.675 |  | -0.007(-0.039, 0.025) | 0.662 |  | -0.100(-0.179, -0.022) | **0.012** |  | -0.033(-0.122, 0.055) | 0.461 |  | -0.031(-0.098, 0.036) | | 0.363 |
| 3-hydroxyphenanthrene | -0.023(-0.048, 0.002) | 0.072 |  | -0.008(-0.035, 0.019) | 0.555 |  | -0.078(-0.143, -0.013) | **0.019** |  | -0.018(-0.091, 0.056) | 0.638 |  | -0.055(-0.111, 0.001) | | 0.053 |
| 4-hydroxyphenanthrene | 0.007(-0.008, 0.022) | 0.362 |  | 0.008(-0.008, 0.024) | 0.320 |  | 0.007(-0.032, 0.046) | 0.724 |  | 0.020(-0.024, 0.063) | 0.380 |  | 0.007(-0.026, 0.040) | | 0.678 |
| 9-hydroxyphenanthrene | 0.035(0.005, 0.065) | **0.021** |  | 0.030(-0.002, 0.062) | 0.062 |  | -0.006(-0.084, 0.073) | 0.888 |  | 0.040(-0.048, 0.127) | 0.377 |  | 0.045(-0.021, 0.112) | | 0.183 |
| ΣOH-PAHs | 0.017(-0.030, 0.064) | 0.480 |  | 0.014(-0.035, 0.064) | 0.570 |  | -0.063(-0.185, 0.058) | 0.306 |  | 0.008(-0.128, 0.144) | 0.907 |  | 0.015(-0.088, 0.119) | | 0.774 |
| ^a^Parameters were ln-transformed prior to inclusion in the analysis.   | ^b^Multivariate linear regression analysis with adjustment for age, gender, BMI, smoking status, pack-years of smoking, alcohol use status, working years, and exercise. | | --- | | | | | | | | | | | | | | | | |

| **Table S5. Associations of urinary PAH metabolites (without creatinine-standardized) with cytokines expression levels (as the dependent variable)** | | | | | | | | | | | |
| --- | --- | --- | --- | --- | --- | --- | --- | --- | --- | --- | --- |
|  | **BDNF^a^** | |  | **ALCAM^a^** | |  | **CRP^a^** | |  | **MSP^a^** | |
| **Variable^a^** | ***β*(95%CI)** | ***P^b^*** |  | ***β*(95%CI)** | ***P^b^*** |  | ***β*(95%CI)** | ***P^b^*** |  | ***β*(95%CI)** | ***P^b^*** |
| 1-hydroxypyrene | -0.106(-0.256, 0.043) | 0.162 |  | 0.043(-0.011, 0.097) | 0.114 |  | 0.304(0.149, 0.459) | **<0.001** |  | 0.055(-0.045, 0.155) | 0.278 |
| 1-hydroxynaphthalene | -0.017(-0.160, 0.127) | 0.820 |  | 0.041(-0.011, 0.093) | 0.121 |  | 0.102(-0.049, 0.253) | 0.187 |  | -0.002(-0.098, 0.094) | 0.965 |
| 2-hydroxynaphthalene | -0.020(-0.160, 0.120) | 0.781 |  | 0.025(-0.025, 0.076) | 0.326 |  | 0.145(-0.002, 0.293) | 0.053 |  | -0.064(-0.158, 0.030) | 0.181 |
| 2-hydroxyfluorene | -0.035(-0.175, 0.105) | 0.626 |  | 0.053(0.003, 0.104) | **0.040** |  | 0.149(0.002, 0.296) | **0.047** |  | -0.024(-0.118, 0.070) | 0.612 |
| 9-hydroxyfluorene | -0.054(-0.104, -0.004) | **0.034** |  | 0.007(-0.011, 0.025) | 0.445 |  | -0.025(-0.078, 0.028) | 0.351 |  | 0.000(-0.034, 0.034) | 0.998 |
| 1-hydroxyphenanthrene | 0.011(-0.080, 0.102) | 0.809 |  | 0.008(-0.025, 0.041) | 0.643 |  | 0.112(0.017, 0.208) | **0.021** |  | 0.038(-0.023, 0.099) | 0.219 |
| 2-hydroxyphenanthrene | 0.079(-0.044, 0.202) | 0.206 |  | 0.064(0.020, 0.108) | **0.005** |  | 0.128(-0.001, 0.257) | 0.051 |  | 0.011(-0.071, 0.094) | 0.787 |
| 3-hydroxyphenanthrene | 0.042(-0.049, 0.133) | 0.364 |  | 0.030(-0.003, 0.063) | 0.075 |  | 0.054(-0.042, 0.150) | 0.268 |  | -0.035(-0.096, 0.026) | 0.263 |
| 4-hydroxyphenanthrene | -0.052(-0.105, 0.000) | 0.051 |  | -0.001(-0.020, 0.018) | 0.936 |  | -0.014(-0.069, 0.041) | 0.617 |  | -0.003(-0.038, 0.033) | 0.887 |
| 9-hydroxyphenanthrene | -0.027(-0.144, 0.090) | 0.650 |  | 0.024(-0.018, 0.067) | 0.259 |  | 0.072(-0.051, 0.196) | 0.249 |  | 0.058(-0.020, 0.137) | 0.146 |
| ΣOH-PAHs | -0.091(-0.298, 0.115) | 0.386 |  | 0.061(-0.014, 0.135) | 0.110 |  | 0.304(0.088, 0.520) | **0.006** |  | 0.028(-0.111, 0.166) | 0.694 |
| ^a^parameters were ln-transformed prior to inclusion in the analysis. | | | | | | | | | | | |
| Regression coefficients were adjusted for age, gender, BMI, smoking status, pack-years of smoking, alcohol use status, working years, and exercise. | | | | | | | | | | | |
|

| **Table S6. Partial correlation coefficient^a^ among PAHs metabolites and ΣOH-PAHs** | | | | | | | | | | | |
| --- | --- | --- | --- | --- | --- | --- | --- | --- | --- | --- | --- |
| PAH metabolites^b^ | 1-hydroxy-pyrene | 1-hydroxy- naphthalene | 2-hydroxy- naphthalene | 2-hydroxy-fluorene | 9-hydroxy-fluorene | 1-hydroxy- phenanthrene | 2-hydroxy- phenanthrene | 3-hydroxy- phenanthrene | 4-hydroxy- phenanthrene | 9-hydroxy- phenanthrene | ΣOH-PAHs |
|
| 1-hydroxypyrene | 1.000 | 0.651^**^ | 0.509^**^ | 0.464^**^ | 0.144^**^ | 0.666^**^ | 0.641^**^ | 0.353^**^ | 0.109^**^ | 0.619^**^ | 0.844^**^ |
| 1-hydroxynaphthalene |  | 1.000 | 0.773^**^ | 0.613^**^ | 0.189^**^ | 0.547^**^ | 0.609^**^ | 0.451^**^ | 0.103* | 0.559^**^ | 0.818^**^ |
| 2-hydroxynaphthalene |  |  | 1.000 | 0.554^**^ | 0.060 | 0.481^**^ | 0.438^**^ | 0.349^**^ | 0.034 | 0.396^**^ | 0.687^**^ |
| 2-hydroxyfluorene |  |  |  | 1.000 | 0.307^**^ | 0.371^**^ | 0.554^**^ | 0.498^**^ | 0.204^**^ | 0.391^**^ | 0.718^**^ |
| 9-hydroxyfluorene |  |  |  |  | 1.000 | 0.127* | 0.071 | -0.090 | 0.369^**^ | 0.184^**^ | 0.388^**^ |
| 1-hydroxyphenanthrene |  |  |  |  |  | 1.000 | 0.474^**^ | 0.195^**^ | 0.062 | 0.539^**^ | 0.686^**^ |
| 2-hydroxyphenanthrene |  |  |  |  |  |  | 1.000 | 0.627^**^ | 0.064 | 0.656^**^ | 0.676^**^ |
| 3-hydroxyphenanthrene |  |  |  |  |  |  |  | 1.000 | 0.016 | 0.342^**^ | 0.441^**^ |
| 4-hydroxyphenanthrene |  |  |  |  |  |  |  |  | 1.000 | 0.106^*^ | 0.264^**^ |
| 9-hydroxyphenanthrene |  |  |  |  |  |  |  |  |  | 1.000 | 0.677^**^ |
| ΣOH-PAHs |  |  |  |  |  |  |  |  |  |  | 1.000 |
| ^a^ Pearson partial correlation with adjustment for Regression coefficients were adjusted for age, gender, BMI, smoking status, pack-years of smoking, alcohol use status, working years, and exercise; ^b^ln-transformed prior to inclusion in the analysis. | | | | | | | | | | | |
|
| ^*^*p* < 0.05 and ^**^*p* < 0.001. | | | | | | | | | | | |
